# Can Hindered Transport Models for Rigid Spheres Predict the Rejection of Single Stranded DNA from Porous Membranes?

**DOI:** 10.3390/membranes12111099

**Published:** 2022-11-04

**Authors:** Hossein Nouri Alavijeh, Ruth E. Baltus

**Affiliations:** 1 Department of Chemical Engineering, University of Virginia, Charlottesville, VA 22904-4259, USA; 2 Department of Chemical and Biomolecular Engineering, Clarkson University, Potsdam, NY 13699-5705, USA

**Keywords:** hindered transport modeling, membrane filtration, DNA filtration

## Abstract

In this paper, predictions from a theoretical model describing the rejection of a rigid spherical solute from porous membranes are compared to experimental results for a single stranded DNA (ssDNA) with 60 thymine nucleotides. Experiments were conducted with different pore size track-etched membranes at different transmembrane pressures and different NaCl concentrations. The model includes both hydrodynamic and electrostatic solute–pore wall interactions; predictions were made using different size parameters for the ssDNA (radius of gyration, hydrodynamic radius, and root mean square end-to-end distance). At low transmembrane pressures, experimental results are in good agreement with rejection predictions made using the hard sphere model for the ssDNA when the solute size is described using its root mean square end-to-end distance. When the ssDNA size is characterized using the radius of gyration or the hydrodynamic radius, the hard sphere model under-predicts rejection. Not surprisingly, the model overestimates ssDNA rejection at conditions where flow induced elongation of the DNA is expected. The results from this study are encouraging because they mean that a relatively simple hindered transport model can be used to estimate the rejection of a small DNA from porous membranes.

## 1. Introduction

A large number of biomolecules, including therapeutic proteins, natural protein products, industrial enzymes, and diagnostic antibodies, are purified and concentrated using ultrafiltration membranes. Over the past several decades, there have been significant advances in the development of gene therapy and other DNA-based pharmaceuticals [1,2,3,4]. Concomitant with these advances, increasing attention has been directed toward DNA purification techniques, including membrane separations [5,6,7].

A number of scholars have sought to estimate membrane performance at the nano- and microscales, with efforts dating back several decades [8,9,10,11,12]. Analytical modeling of a membrane filtration process is a difficult problem, since calculations must account for the impact of mechanical, chemical, and electrical phenomena on flow across the membrane [8]. Theoretical models for hard spheres that account for the steric, hydrodynamic, and long-range (electrostatic) interactions between the solute and the pore have generally been successful in describing protein transport through semipermeable membranes [13,14,15,16,17].

The degree of separation in porous membranes is often described using the rejection coefficient, *R*, which is defined by [9]:

(1) $$R=1-{\left[\frac{N_{s}}{C_{b}J_{v}}\right]}_{∆C_{b}=0}$$

where $N_{s}$ and $J_{v}$ are the solute and solvent flux across the membrane, respectively, and $C_{b}$ is the concentration of solute in the bulk feed.

**Figure 1:**
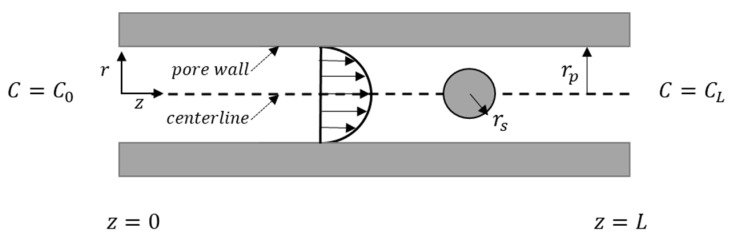
Schematic 2D diagram of a rigid spherical particle in a cylindrical pore.

Previous hindered transport modeling has often treated the solute as a Brownian particle and has largely considered particles of simple shape (i.e., spheres) traversing pores of simple and uniform cross section, as shown in Figure 1, for a spherical solute in a cylindrical pore. When convection is the primary means of particle or solute transport through the porous membrane, these models have generally begun with the following definition of the filtration rejection coefficient [18], which incorporates both solute partitioning between pore and bulk solutions and hydrodynamic resistances experienced by the solute in the pore:

(2) $$R=1-\frac{\int G\left(X\right)v\left(X\right)\exp\left(-\frac{E\left(X\right)}{k_{B}T}\right)\;dX}{\int v\left(X\right)dX}$$

Here, X(x,y) is the two-dimensional position vector in the pore, E(X) is the particle–pore wall interaction energy, G(X) is the local lag coefficient, which characterizes the hydrodynamic interactions (defined as the velocity of the particle at position X, relative to the fluid velocity at X if the particle was not present), v(X) is the solution velocity in the pore, $k_{B}$ is the Boltzmann constant, and T is temperature. The hydrodynamic interactions are altered by any force that impacts the particle’s position in the pore. Even if long-range forces (e.g., van der Waals or electrostatic forces) are insignificant, the solute’s finite size limits its access to the region near the wall, affecting its flow [13]. Equation (2) assumes solute–solute interactions are negligible.

Early theoretical efforts to describe G(X), as well as the analogous hindrance factor for diffusion, were summarized in a review article by Deen [10]. More recently, Dechadilok and Deen [13] provided an updated review that includes more accurate expressions for both convective and diffusive hindrance factors for rigid spherical particles. When solute and membrane are charged, Dechadilok and Deen [19] demonstrated that electrokinetic effects on G are generally insignificant.

When electrostatic solute–pore wall interactions can be neglected, the Boltzmann term in Equation (2) ($\exp\left(-E\left(X\right)/\left(k_{B}T\right)\right)$) has a value of either 0 or 1, depending upon whether or not the solute can be placed at position X in the pore. When electrostatic solute–membrane interactions are important, the Poisson–Boltzmann equation (PB) must be solved for the electrical potential profile in the pore, from which E can be determined:

(3) $$\nabla^{2}\psi =\tau^{2}sinh\left(\psi \right)$$

where ψ is the dimensionless electrostatic potential and τ is the dimensionless inverse Debye length. For a 1-1 salt, τ is defined by:

(4) $$\tau =ϒk_{D}^{-1}=ϒ\;{\left(\frac{2F^{2}}{\varepsilon RT}C\right)}^{1/2}$$

Here,$\;k_{D}$ is the Debye length, ϒ is the appropriate length scale (pore radius or solute radius), F is the Faraday constant (96,485.33 C/mol), C is the salt concentration, and ε is the dielectric permittivity. When the electrostatic energy is small compared to the thermal energy, one can assume sinh(ψ)≈ψ and Equation (3) can be simplified to its linear form:

(5) $$\nabla^{2}\psi =\tau^{2}\psi$$

To determine E(X) in Equation (2), the PB equation must be solved for three situations: the electrostatic potential for a sphere in a pore, for an unbounded sphere, and for a cylindrical pore free of the sphere [14]. The potential energy for each situation is then determined by integrating ψ over the appropriate surface. The interaction energy in Equation (2) is then calculated by subtracting the potential energy for the unbounded sphere and for the cylindrical pore from the potential energy for the sphere in the pore.

Smith and Deen [14] presented solutions to Equation (5) for an unbounded sphere, for a cylindrical pore, and a centerline (X=0) solution for a spherical particle in a cylindrical pore. Burns and Zydney [17] presented the Smith and Deen solution for E(0) in a convenient form:

(6) $$\frac{E\left(0\right)}{k_{B}T}=\frac{A_{s}\sigma_{s}^{2}+A_{p}\sigma_{p}^{2}+A_{s}\sigma_{s}\sigma_{p}}{A_{den}}\;\;$$

where $\sigma_{s}$ and $\sigma_{p}\;$are the dimensionless surface charge densities on the solute and pore wall, respectively, and $A_{s\;},A_{p\;},A_{sp\;}$ and $A_{den\;}$are all positive coefficients that depend on the solution ionic strength, pore radius, and solute radius. Expressions for the coefficients $A_{s\;},A_{p\;},A_{sp\;,}$ and $A_{den\;}$are listed in Table S1 in the Supplementary Materials.

There have been numerous studies examining membrane filtration of rigid particles, proteins, DNA, and other flexible molecules using both experimental and modeling approaches. Agasanapura et al. [18] examined the rejection of rigid spheres and rigid rods from polycarbonate track-etched porous membranes with both low and high ionic strength solutions. A model based on Equation (2) was developed, which included electrostatic particle–membrane interactions, as well as the effect of particle orientation on hydrodynamic resistances. Experimental results were in good agreement with model predictions for both low and high ionic strength conditions. Delavari et al. [20] examined the rejection of polystyrene latex particles from virgin track-etched membranes, which carry a negative charge, as well as from surface modified track etched membranes, which had net zero charge. Results were in good agreement with Equation (2) when particle adsorption to the membrane surface was considered.

Results from a number of studies investigating protein rejection by porous membranes have shown protein transport to be dependent on solution pH and ionic strength, as well as on membrane surface charge, demonstrating the importance of electrostatic solute–membrane interactions in these systems [16,17,21,22]. Models of these systems have generally followed the framework of Equation (2), where the proteins are modeled as rigid impermeable spheres with uniform surface charge distribution. Under most conditions, the important factor governing protein transport is the protein partitioning between pore and bulk solutions controlled by the electrostatic protein–membrane interactions.

Others have developed models for predicting the rejection of linear flexible molecules from small pores [23,24,25,26]. Davidson et al. [25] employed a Monte Carlo technique to predict the partition coefficient of freely jointed chains between a cylindrical pore and bulk solution, showing that the partition coefficient for a freely jointed chain is smaller than that of a rigid sphere with the same hydrodynamic (i.e., Stokes–Einstein) radius. Davidson and Deen [27] developed a model for the hindered transport of flexible macromolecules in porous membranes. This model did not consider any electrostatic interactions and combined the partitioning model reported earlier by Davidson et al. [25] with a model of the hydrodynamic resistances experienced by a porous sphere traveling in a small pore. Although the hydrodynamic resistance experienced by a porous sphere is predicted to be less than that of an impermeable sphere, the smaller partition coefficient for the freely jointed chain leads to a lower convective hindrance factor (which is essentially a sieving coefficient = 1−R) for the flexible macromolecule, compared to the solid sphere. In terms of rejection, their model predicts a larger rejection coefficient for the flexible solute than for a solid sphere with the same hydrodynamic radius.

Morao et al. [24] developed a stochastic model based on the Davidson et al. [25] approach to determine the partition coefficient of a flexible molecule (modeled as a freely jointed chain) much larger than the pore, which led to an expression for the rejection coefficient. Experimental results for the filtration of T2000 dextran and a linear DNA were in reasonable agreement with model predictions. These models indicate that models for rigid spherical particles do not apply for large flexible molecules, particularly those with molecular weight exceeding 1 MDa and radius of gyration larger than 10 nm [6,24].

There have been numerous studies focused on DNA transmission across ultrafiltration membranes [14,18,21,22,23,24]. Results from these studies have demonstrated the importance of filtrate flux, observations that have been attributed to flux induced elongation. Latulippe and Zydney [28] proposed a flow-induced elongation model that is based on a free draining model for the DNA and incorporates the flexibility of the DNA through its persistence length. The model predicts the critical flux, defined as the flux below which DNA transmission is negligible and above which it is significant. Experimental results reported by Latulippe and Zydney [28], as well as results reported by our laboratory [29] for a 60 nucleotide single-stranded DNA, were in general agreement with the critical flux predicted by the model. Previous studies have also shown DNA and RNA transmission rates to increase as solution ionic strength is increased [29,30,31,32,33]. These trends can be attributed to decreased solute size, as well as decreased repulsive electrostatic solute–membrane interactions as ionic strength is increased. These previous studies have contributed to an increased understanding of the parameters important in DNA filtration. However, efforts to develop quantitative predictions of DNA rejection or transmission across porous membranes have been limited.

We have recently reported results from an experimental study examining the rejection of a single stranded DNA (ssDNA) with 60 thymine nucleotides (poly(dT_60_)) from polycarbonate track-etched membranes [29,34]. With appropriate assumptions, it is straightforward to use Equation (2) to estimate solute rejection for a rigid sphere. The objective of the work presented in this paper was to examine the validity of such a model to quantitatively predict rejection for poly(dT_60_). This model includes both electrostatic and hydrodynamic ssDNA–membrane interactions and considered predictions developed using different parameters to describe ssDNA size (hydrodynamic radius, radius of gyration, and root mean square end-to-end distance).

## 2. Materials and Methods

We begin with a description of the details and results from the previously reported experimental study [29,34].

### 2.1. Experiment

The poly(dT_60_) used in the filtration experiments was synthesized by and purchased from Integrated DNA Technology Inc. (IDT, Coralville, IA, USA). Poly(dT) is an important and widely studied model system for flexible single stranded nucleic acids [35,36,37,38,39,40,41,42]. This ssDNA is a flexible homopolymer that does not have base pairing capabilities, behaves as a linear fragment [38,42], and has a polydispersity index of 1 [34].

The filtration experiments were conducted using membranes with 10 nm, 15 nm, and 30 nm nominal pore sizes, in pH 8 IDTE buffer (10 mM Tris-HCl, 0.1 mM EDTA) with NaCl concentration ranging from 0 mM to 100 mM at transmembrane pressures (TMP) of 69 kPa (10 psi), 138 kPa (20 psi), and 207 kPa (30 psi). The IDTE buffer adds −5 mM to the ionic strength of these solutions. Streaming potential measurements were used to determine the zeta potential of the pore surface, yielding −68 mV for the 10 nm membranes, −49 mV for the 15 nm membranes, and −38 mV for the 30 nm membranes. The track-etched membranes have generally uniform cylindrical pores, making it straightforward to compare experimental results to model predictions. Membrane pore size was determined before and after each filtration experiment using hydraulic permeability measurements, showing no changes in pore size. Scanning electron microscopy (SEM) imaging of the membrane surface revealed circular pores (see Figure S1 in the Supplementary Materials). SEM imaging was also used to determine membrane thickness, yielding values ranging from 3.8 μm to 5.3 μm (depending on membrane pore size) [34]. With a pore length/pore diameter ratio greater than 100 for all membranes, pore entrance and exit effects can be neglected in transport modeling.

Filtration experiments involved measuring the poly(dT_60_)concentration in the permeate ($C_{p}$) and feed ($C_{b}$) using UV absorbance at 260 nm [29,34]. The ssDNA concentration in the feed was 20 ppm, providing conditions consistent with an infinite dilution assumption. Stir speed in the filtration cell was sufficiently high (480 rpm) so that concentration polarization could be neglected [34]. Poly(dT_60_) rejection was then calculated from the measured concentrations using:

(7) $$R\;\left(\%\right)=100\times \left[1-\frac{C_{p}}{C_{b}}\right]$$

A design of experiments methodology was used to design 18 filtration experiments, yielding poly(dT_60_) rejections ranging from 10.3% to nearly complete rejection depending on pore size, TMP, and ionic strength. The experimental conditions and rejection results from these 18 experiments are listed in Table S2 in the Supplementary Information. The average experimental uncertainty was 7.4%. An empirical expression was developed from the results, relating ssDNA rejection to pore size in nm ($D_{p}$), NaCl concentration in mM (C), and transmembrane pressure in psi (*TMP*):

(8) $$R={\left(9.89+0.0160\;D_{p}+0.0762\;TMP-0.0388\;C-0.00624\;D_{p}\times TMP-0.00143\;D_{p}\times C+0.000389\;C^{2}\right)}^{2}$$

A comparison of predictions from Equation (8) to experimental values (including results from 7 additional experiments that were not used in developing Equation (8)) revealed excellent agreement, with a parity plot yielding a coefficient of determination ($R^{2}$) of 0.94 [29,34] (see Figure S2 in the Supplementary Materials). Static poly(dT_60_) uptake measurements showed insignificant ssDNA adsorption to the membrane surface [34].

Results showed expected trends with pore size and ionic strength, with rejection increasing with decreasing $D_{p}$ and decreasing C (because of increasing repulsive ssDNA–membrane interactions). Rejections from the smaller pore membranes were generally insensitive to TMP (i.e., filtrate flux). A significant decrease in rejection was observed with the 30 nm pore size membranes with increasing TMP; our analysis showed that this decrease in rejection is due to flow-induced elongation of the ssDNA under high flux conditions [29].

The size and flexibility of the poly(dT_60_) was determined from diffusion coefficient values measured under different ionic strength conditions by fluorescence recovery after photobleaching in the Zipfel Lab at Cornell University. The zeta potential of the ssDNA was measured using a Zetasizer Nano particle analysis instrument, yielding −38 mV at pH 8.

The Stokes–Einstein equation was used to calculate the hydrodynamic radius ($R_{h}$) of the poly(dT_60_) from its diffusivity, *D*:

(9) $$R_{h}=\frac{k_{B}T}{6\pi \mu D}$$

where μ is the solution viscosity. A worm-like chain (WLC) model was used to determine the radius of gyration, $R_{G}$, from $R_{h}$ [43]:

(10) $$\frac{R_{h}}{R_{G}}≅\frac{2\sqrt{12}}{\ln\left(M\right)}$$

where M is the molecular weight. The molecular weight of poly(dT_60_) ssDNA is 18728 Da, yielding, *R_h_/R_G_* = 0.704.

The root mean square end-to-end distance ($\langle h^{2}\rangle {}^{1/2}$) of a WLC chain is related to the contour length (L), which is equal to the number of monomers or base pairs times and the distance per monomer (0.43 nm for single-stranded DNA [43,44,45,46], giving L=27 nm here) and persistence length ($l_{p}$) by [47]:

(11) $$\langle h^{2}\rangle {}^{1/2}={\left(2l_{p}L\right)}^{1/2}{\left[1-\frac{l_{p}}{L}+\frac{l_{p}}{L}\;\exp\left(-\frac{L}{l_{p}}\right)\;\right]}^{1/2}$$

The persistence length represents the degree of flexibility of a polymer chain and was determined from $R_{G}$ using the WLC model [48]:

(12) $$R_{G}=l_{p}{\left[\frac{L}{3l_{p}}-1+\frac{2l_{p}}{L}-2{\left(\frac{l_{p}}{L}\right)}^{2}\left(1-\exp\left(-\frac{L}{l_{p}}\right)\right)\right]}^{1/2}$$

The values of D measured in different NaCl concentrations and the calculated values of *R_h_*, *R_G_*, $\langle h^{2}\rangle {}^{1/2},$ and $l_{p}$ are summarized in Table 1.

### 2.2. Modeling

#### 2.2.1. Solute and Pore Sizes

Ultrafiltration separations of proteins have been successfully modeled using hard sphere solute models [16,17,21,22,49]. The objective of the work reported here was to examine the validity of a hard sphere model for a small flexible solute, such as poly(dT_60_) ssDNA. To predict rejection using Equation (2), values for the lag coefficient, *G*, and the interaction energy, *E*, are required. Both parameters require estimates of the solute size. Unlike a rigid sphere, different size parameters can be used to describe the size of a flexible solute, such as ssDNA. Here, model predictions were developed using $R_{h}$, $R_{G}$ and $\langle h^{2}\rangle {}^{1/2}$. Membrane pore size values determined from hydraulic permeability measurements performed before and after each filtration experiment were used in the model calculations [34].

#### 2.2.2. Hydrodynamic Interactions

The lag coefficient G is related to the ratio of solute to pore size, as well as the solute position in the pore. Higdon and Muldowney [50] used a spectral boundary element method with high-order discretization to formulate the resistance coefficients for low Reynolds number flow past a spherical particle at different radial positions in a cylindrical pore. For our problem, two coefficients are important: $R_{z}$ is the resistance coefficient for a sphere moving in the axial direction in a pore with fluid moving in parabolic flow and $R_{p}$ is the resistance coefficient for a sphere held fixed in a parabolic flow field. Both are functions of sphere and pore size. Higdon and Muldowney [50] developed convenient algebraic functions for $R_{z}$ and $R_{p}$ from their numerical results, making it straightforward for us to use their results in our rejection model. Combining an expression from Deen [10] that relates the hydrodynamic force experienced by a solute to G with an analogous expression presented by Higdon and Muldowney [50] that relates the hydrodynamic force to the resistance coefficients $R_{z}$ and $R_{p}$ yields [34]:

(13) $$G=\frac{R_{p}}{R_{z}\left[1-{\left(\frac{r}{r_{p}}\right)}^{2}\right]}$$

Calculations [13,34] have shown that G is nearly independent of pore position. Therefore, a centerline approximation, G(r=0), was used in all of our calculations.

#### 2.2.3. Electrostatic Interactions

The nonlinearized PB equation (Equation (3)) was solved using COMSOL-Multiphysics 5.4 in the 3D domain, using the following boundary conditions for both the sphere and the pore wall:

(14) ψ|surface=ψ0e(kBT)

(15) $${\left(\nabla \psi \right)\cdot \hat{n}|}_{surface}=\sigma$$

where $\psi_{0}$ is the surface potential (assumed to equal the measured zeta potential), e is the electron charge (1.609 × 10^−19^ C), σ is the dimensionless surface charge density, and $\hat{n}$ is the outward unit normal vector to the surface.

The solution to the linearized PB equation (Equation (6)) requires values for the surface charge densities of the DNA and the pore wall. These were calculated from the measured zeta potentials using Equations (S2)–(S5) provided in the Supplementary Materials.

## 3. Results and Discussion

Our objective in this study was to determine the validity of a hard sphere model for describing the filtration of a small ssDNA, poly(dT_60_). Electrostatic and hydrodynamic interactions between the solute and the membrane pore walls were included in the model calculations. Here, we begin by presenting some modeling results, illustrating the effect of pore size and salt concentration on solute partitioning and rejection. Comparisons of some experimental observations to model predictions are then presented and discussed.

### 3.1. Linearized vs. Non-Linearized Poisson Boltzmann Equation

**Figure 2:**
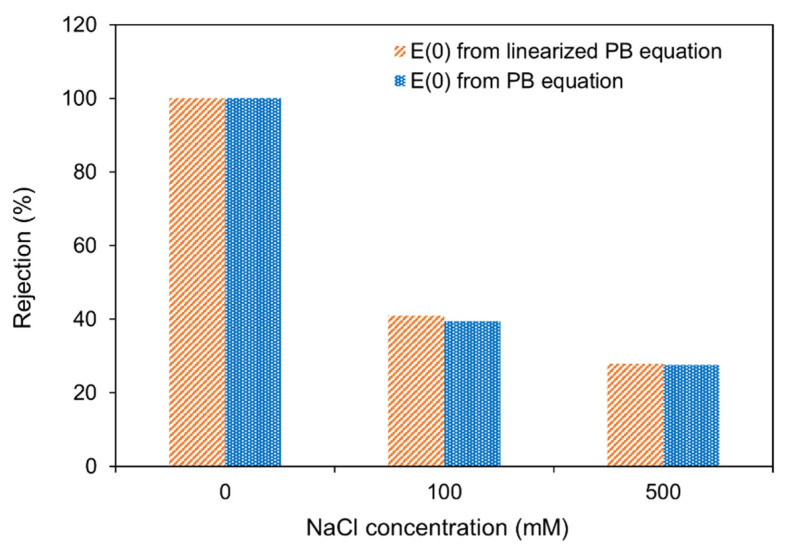
Comparison of rejection predictions for poly(dT_60_) ssDNA made using the non-linear PB equation (solution of Equation (3)) to predictions made using the solution of the linearized PB equation (Equation (5)) to describe electrostatic DNA–membrane interactions for a 30 nm pore membrane. Hydrodynamic interactions were neglected in these calculations (i.e., G=1).

The rejection of a rigid spherical solute from a cylindrical pore at different ionic strength conditions was computed using Equation (2), where E(0) was estimated using both the PB equation, as well as its linearized form (Equations (3) and (5)), to describe the electrostatic solute–pore wall interactions. Here, the potential on the spherical solute and on the pore wall were assumed equal to the measured zeta potentials of the ssDNA and the polycarbonate membrane. A comparison of the predicted rejections is presented in Figure 2, showing excellent agreement between the two solution approaches. Therefore, in subsequent modeling, the solution of the linearized PB equation (Equation (6)) was used to calculate E(0) for Equation (2).

### 3.2. Hydrodynamic Interactions

**Figure 3:**
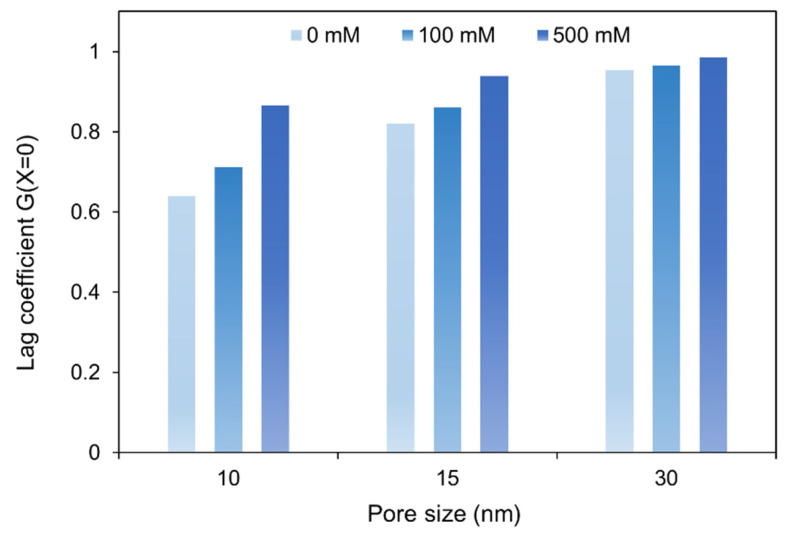
Model predictions for the centerline lag coefficient of poly(dT_60_) ssDNA with effective size of $R_{G}$ in different membrane pore sizes at different NaCl concentrations.

The centerline lag coefficient, G(0), was calculated using the analytical expression developed by Higdon and Muldowney [50] (Equation (13)). In Figure 3, the lag coefficient of poly(dT_60_) ssDNA in different membrane pore sizes for three different NaCl concentrations are compared. The radius of gyration of poly(dT_60_) ssDNA was used as the solute size in these calculations.

In these calculations, electrokinetic effects were neglected. Therefore, the impact of ionic strength on the lag coefficient arises solely through the dependence of poly(dT_60_) ssDNA size on ionic strength (Table 1). Hydrodynamic resistances experienced by the ssDNA are negligible in the 30 nm pore size membrane; therefore, the lag coefficient is generally insensitive to ionic strength. As pore size decreases, the hydrodynamic interactions become more significant and the sensitivity of G to ionic strength increases.

### 3.3. Poly(dT_60_) ssDNA Rejection: Model Predictions and Experimental Results

As reported in [29] and noted here before, a design of experiments approach was used to plan and perform 18 filtration experiments, covering a range of NaCl concentrations, membrane pore sizes, and transmembrane pressures. Results from these experiments were analyzed to develop the correlation in Equation (8). Here, we use rejection values calculated from this correlation to assess the validity of our model predictions. In the discussion to follow, we use ‘experimental’ (in quotes) when referring to rejection values estimated from Equation (8).

To calculate ssDNA rejection from the hard sphere model (Equation (2)) requires the size of the poly(dT_60_) at the ionic strength under consideration. The poly(dT_60_) ssDNA diffusion measurements were limited to the solutions listed in Table 1. In order to estimate the poly(dT_60_) ssDNA size in solutions with other NaCl concentrations, the hydrodynamic radii determined from the measured diffusivities in 0 mM, 100 mM, and 500 mM NaCl solutions were fitted to a power law expression, yielding:

(16) $$R_{h}=2.82-0.01\;C^{0.75}$$

where $R_{h}$ is in nm and C is the NaCl concentration in mM. A comparison of $R_{h},$ calculated from the measured diffusivities to Equation (16) is shown in the Supplementary Materials (Figure S3), showing a very good fit.

**Figure 4:**
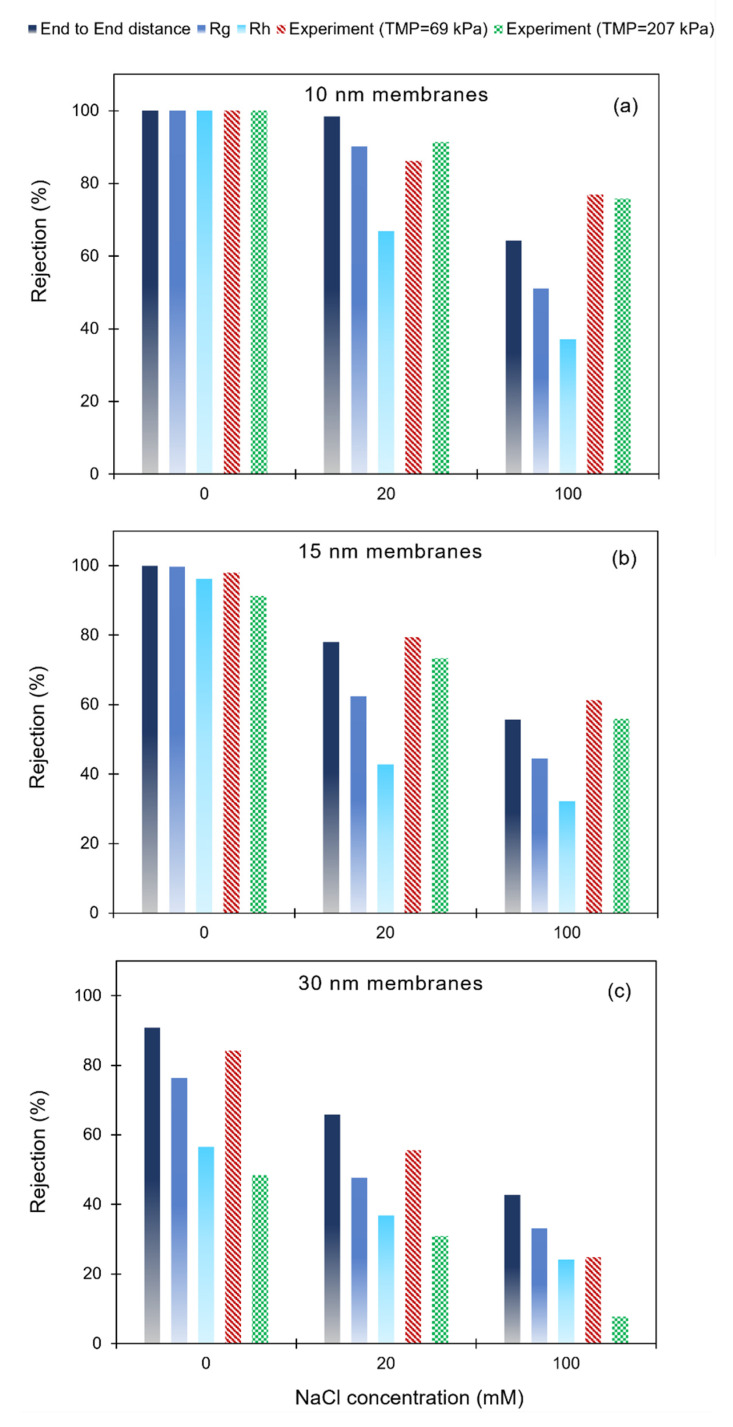
Comparison of model predictions to ‘experimental’ values for poly(dT_60_) ssDNA rejection from 10 nm pore size membranes (**a**), 15 nm pore size membranes (**b**) and 30 nm pore size membranes (**c**). Model predictions were determined using different ssDNA size parameters ($\langle h^{2}\rangle {}^{1/2}$, $R_{G}$, and $R_{h}$. For the 0 mM and 100 mM NaCl concentration solutions, ssDNA sizes were determined from measured diffusivities and Equations (9)–(11) (Table 1). For the 20 mM NaCl concentration solutions, $R_{h}$ was determined using Equation (16), and $R_{G}$ and $\langle h^{2}\rangle {}^{1/2}$ were determined using Equations (10) and (11).

Comparisons of model predictions for poly(dT_60_) rejection to ‘experimental’ results are presented in Figure 4a for 10 nm pore size membranes, in Figure 4b for 15 nm pore size membranes, and in Figure 4c for 30 nm pore size membranes. Here, we present ‘experimental’ results for measurements at 69 kPa (10 psi) and 207 kPa (30 psi) TMP. The model predictions were developed using different size parameters for the ssDNA: $R_{h}$ (hydrodynamic radius), $R_{G}$ (radius of gyration), and $\langle h^{2}\rangle {}^{1/2}$ (root mean square end-to-end distance). For these calculations, we considered $\langle h^{2}\rangle {}^{1/2}$ to be characteristic of the sphere diameter.

This discussion will first focus on the effect of TMP on ‘experimental’ ssDNA rejection. Poly(dT_60_) rejections for filtration measurements performed at 69 kPa and 207 kPa TMP are compared for each membrane in Figure 4. The average experimental uncertainty in the rejection results was 7.4%. The small differences in ‘experimental’ ssDNA rejections from 10 nm pore size membranes and 15 nm pore size membranes at 69 kPa and 207 kPa are less than this uncertainty. Therefore, one can conclude that the ssDNA rejections are independent of TMP at all NaCl concentration for these membranes. This indicates that the size and conformation of the ssDNA is generally independent of TMP for these systems.

In contrast, results show that the ‘experimental’ rejections from the 30 nm pore size membranes are considerably larger for experiments conducted at 69 kPa, compared to experiments conducted at 207 kPa. This dependence on TMP can be attributed to the flux-induced elongation of the ssDNA at the higher TMP, resulting in larger ssDNA transmissions across the membrane (i.e., smaller rejection). Previous analyses have shown that elongation of flexible solutes can occur at fluxes larger than the critical flux [28,29]. Therefore, it is reasonable to expect the conformation of a flexible solute to be different at conditions above the critical flux than below this flux.

A free draining model developed by Latulippe and Zydney [28] was used to estimate the critical flux for these measurements from ssDNA size, membrane pore size, and membrane porosity, yielding a critical flux of 31 L/(m^2^ h) for the 30 nm pore size membranes challenged with poly(dT_60_) [29]. Experiments conducted at 207 kPa with the 30 nm pore size membranes had flux values larger than 50 L/(m^2^ h), indicating that the ssDNA was subject to flux-induced elongation at this TMP. At 69 kPa TMP, flux values were all considerably smaller than the critical flux. Flux values for experiments with the 10 nm and 15 nm pore size membranes were all much smaller than the estimated critical flux for these systems, even at a TMP of 207 kPa.

The ‘experimental’ results presented in Figure 4a show ssDNA rejections decreasing with increasing salt concentration for the 10 nm pore size membrane; all of the model predictions show the same qualitative trend. A total of 100% of rejections were observed and are predicted for the DNA at 0 mM NaCl concentration. The lag coefficient predictions shown in Figure 4 show G in this membrane at these conditions to be ~0.6. The difference between the predicted rejection and the predicted lag coefficient can be attributed to the repulsive electrostatic interactions, which are significant in the low ionic strength solution and lead to reduced partitioning between pore and bulk solutions.

In the higher ionic strength solutions, rejection predictions are largest when $\langle h^{2}\rangle {}^{1/2}$ is used to estimate the solute size and smallest when $R_{h}$ is used as the characteristic solute size. Model predictions based on $\langle h^{2}\rangle {}^{1/2}$ as the characteristic solute size are generally consistent with the ‘experimental’ results. However, model predictions for ssDNA rejection underestimate ‘experimental’ observations when $R_{G}$ and$\;R_{h}$ are used to characterize the ssDNA size.

Model predictions and ‘experimental’ results for 15 nm membranes are presented in Figure 4b, with qualitative trends similar to those seen with the 10 nm membranes. However, electrostatic interactions play a smaller role in the 15 nm membranes, even in the low ionic strength solutions. Again, model predictions for rejection based on$\;\langle h^{2}\rangle {}^{1/2}$ as the characteristic size of the ssDNA are seen to be in general agreement with ‘experimental’ results, whereas rejections predicted using $R_{G}$ or$\;R_{h}$ as the ssDNA size are considerably smaller than observed values.

As noted earlier, experiments performed at a TMP of 207 kPa with the 30 nm pore size membranes yielded fluxes that were larger than the critical flux, leading to elongation of the DNA under these conditions. At TMP = 69 kPa, flux-induced elongation is not expected. Comparing model predictions to the 69 kPa ‘experimental’ results shows good agreement when $\langle h^{2}\rangle {}^{1/2}$ is used as the characteristic ssDNA size for 0 mM and 20 mM NaCl concentrations, in agreement with the comparisons seen with the 10 nm and 15 nm pore size membranes.

These observations are qualitatively consistent with the conclusions drawn from a model developed by Davidson and Deen [27] for the hindered transport of flexible macromolecules in porous membranes. Their model shows that using the hydrodynamic radius as the characteristic size of a flexible solute leads to an under-prediction in rejection, in qualitative agreement with the results from our study. This is not surprising as one might expect a WLC to exhibit reduced partitioning and reduced hydrodynamic resistances relative to a hard sphere with the same hydrodynamic radius, qualitatively similar to the freely jointed chain modeled by Davidson and Deen.

Stochastic simulations were used by Morao et al. [24] to develop a model for the partition coefficient of large linear molecules. Assuming the solute occupies the entire pore cross section (i.e., it travels at the fluid velocity in the pore), the rejection coefficient was then calculated directly from the partition coefficient. Their simulation results were fitted to a sigmoidal function, providing a convenient analytical expression relating the rejection coefficient to the solute and pore size. This expression uses the root mean end-to-end distance of the chain as the characteristic solute size, which is the same as the parameter we found to best describe the hindered transport of the ssDNA represented as a sphere. However, when their analytical expression was used to calculate the rejection of the poly(dT_60_), nearly zero rejection was predicted, even for values measured in high ionic strength solutions where repulsive electrostatic interactions should not be important. As shown in Figure 4, our results showed rejections ranging from ~20% to ~80% (depending on pore size) at high ionic strength.

Both the Davidson and Deen [27] and the Morao et al. [24] models considered solutes that are freely jointed chains. With a contour length of 27 nm and persistence length values ranging from 0.6 nm to 2.2 nm (Table 1), the number of WLC segments characteristic of the poly(dT_60_) is anticipated to range from ~12 to 45, much smaller than expected for a freely jointed chain. Therefore, the fact that the expression developed from the Morao et al. [24] simulations predicts rejections considerably smaller than our experimentally measured values is not particularly surprising. We also note that neither of these previous modeling studies considered other solute size parameters to represent the flexible solutes.

Most of the results from this study show that under flux conditions below the critical flux, one can make reasonable predictions of the solute rejection of small ssDNA by modeling the solute as a hard sphere, where the solute size is characterized using $\langle h^{2}\rangle {}^{1/2}$. Using either $R_{G}$ or$\;R_{h}$ as the solute size results in a considerable under-prediction of rejection. However, when flow-induced elongation is expected (as for the experiments conducted with a TMP of 207 kPa in the 30 nm pore size membranes), rejections predicted based on using $\langle h^{2}\rangle {}^{1/2}$ are much larger than measured values. This is not surprising because one expects a different ssDNA conformation at these conditions.

There is one exception to the trends seen with the 10 nm and 15 nm pore size membranes and with the 30 nm pore size membranes at low NaCl concentrations. Filtration experiments conducted in a 30 nm pore size membrane with the 100 mM NaCl solution at 69 kPa TMP yielded ssDNA rejection in general agreement with the prediction made using $R_{h}\;$as the characteristic ssDNA size and considerably smaller than the rejection predicted using $\langle h^{2}\rangle {}^{1/2}$ as the characteristic size, in contrast to comparisons seen at other conditions.

The Boltzmann factors ($\exp\left(-E\left(0\right)/\left(k_{B}T\right)\right)$) in the three different pore size membranes were all estimated to be close to unity in 100 mM NaCl (0.94 in 10 nm membrane and 0.99 in a 30 nm membrane). Therefore, the primary mechanism contributing to rejection at high NaCl appears to be the hydrodynamic resistances experienced by the ssDNA. Because a different ssDNA size parameter is needed to predict rejection in the 30 nm pore size membrane, compared to the 10 nm and 15 nm membranes, the ssDNA is apparently effectively smaller in the 30 nm membrane than in the smaller pore membranes. This difference only occurs under high ionic strength conditions. The increased flexibility of the ssDNA at 100 mM NaCl may explain why this difference does not arise at lower NaCl conditions. Additional study is warranted to further examine these observations.

It is recognized that ssDNA, such as the poly(dT_60_), examined in this work has a complex conformation that is often modeled as a WLC [37,40,43,44]. However, the fact that the transport of poly(dT_60_) in nanopores follows a hard sphere model was unexpected, yet very convenient from a modeler’s perspective. We speculate that this conclusion may be related to the small size of this solute. Additional studies examining the filtration of longer chain ssDNAs should help to identify the limits of the hard sphere model when describing the hindered transport of these important solutes.

## 4. Conclusions

In this paper, we report comparisons of previously reported experimental results for the rejection of a small ssDNA, poly(dT_60_), from track-etched polycarbonate membranes [29] to predictions made using a hindered transport model, where the ssDNA is assumed to behave as a rigid sphere. The model includes both repulsive electrostatic ssDNA–membrane interactions, as well as hydrodynamic resistances experienced by the DNA, as it transports through the membrane pores. Not surprisingly, the model over-predicts rejection when the TMP is sufficiently high, such that the flux is above the critical flux and the ssDNA experiences flow-induced elongation. However, under most conditions, rejection predictions made using the hard sphere model for the ssDNA are in good agreement with ‘experimental’ results when the solute size is described using its root mean square end-to-end distance. When the ssDNA size is characterized using the radius of gyration or the hydrodynamic radius, the hard sphere model generally under-predicts rejection from the membrane.

Modeling the hindered transport of a WLC in a small pore is a challenging exercise. Therefore, the results from this study can be useful when designing a membrane filtration separation for small DNA. It is fairly straightforward to use Equation (2) to predict rejection for a spherical solute from a porous membrane when solute and membrane pore sizes and their surface characteristics are known. This approach is much simpler than using the WLC model to describe the ssDNA characteristics. However, there is one caveat to this conclusion and that pertains to describing rejection in a relatively large pore at high ionic strength. Under these conditions, our results show that experimentally observed rejections agree with model predictions for a hard sphere solute when the ssDNA size is described using the solute hydrodynamic size, rather than the root mean square end-to-end distance. The reason for this difference is not well understood at this time.

## Figures and Tables

**Table 1:** Properties of poly(dT_60_) ssDNA at different NaCl concentrations [29]. The diffusion coefficient measurements were conducted in pH 8 IDTE buffer solutions.

NaCl Concentration (mM)	Diffusion Coefficient (µm^2^/s)	$R_{h}\;$(nm)	$R_{G}\;$(nm)	$\langle h^{2}\rangle {}^{1/2}\;$(nm)	$l_{p}\;$(nm)
0	85 ± 5	2.8 ± 0.3	4.0 ± 0.5	10.5	2.2
100	96 ± 13	2.5 ± 0.4	3.5 ± 0.6	9.0	1.6
500	142 ± 16	1.6 ± 0.1	2.3 ± 0.2	5.6	0.6
1000	136 ± 10	1.6 ± 0.1	2.2 ± 0.2	5.6	0.6

## Data Availability

Data are available upon request from R. Baltus.

## Supplementary Materials

The following supporting information can be downloaded at: https://www.mdpi.com/article/10.3390/membranes12111099/s1, Figure S1: SEM image of membrane surface for 30 nm pore size membrane. Figure S2: Parity plot of predicted vs. measured rejections. Figure S3: Hydrodynamic radius of poly(dT_60_) ssDNA at different NaCl concentrations from the measured diffusion coefficients. Table S1: Coefficients in the solution of the linearized Poisson Boltzmann equation (Equations (6) and (S1)). Table S2: Results from Filtration Experiments used to develop Equation (8).
