# Supplementary material for: Can Hindered Transport Models for Rigid Spheres Predict the Rejection of Single Stranded DNA from Porous Membranes?
Source: Membranes (Basel). 2022 Nov 4;12(11):1099. doi: 10.3390/membranes12111099 (PMC9694696; doi:10.3390/membranes12111099)
Supplement: Supplementary file 1 [file membranes-12-01099-s001.zip › membranes-1952619-supplementary.pdf]

Supplementary Materials

Can Hindered Transport Models for Rigid Spheres Predict the Rejection of Single Stranded DNA from Porous Membranes?

Hossein Nouri Alavijeh and Ruth Baltus

Linearized Poisson Boltzmann Equation

Equation (6) is the solution of equation (5), the linearized Poisson Boltzmann equation

which is repeated here for clarity:

$$\frac{E(0)}{k_B T} = \frac{A_s\sigma_s^2 + A_p\sigma_p^2 + A_s\sigma_s\sigma_p}{A_{den}}$$	(S1)

where σ_s and σ_p are the dimensionless surface charge densities of the solute and pore wall, respectively, and are defined by

$$\sigma_s = \frac{F.r_p.q_s}{\varepsilon_0\varepsilon_r RT}$$	(S2)
$$\sigma_p = \frac{F.r_p.q_p}{\varepsilon_0\varepsilon_r RT}$$	(S3)

Here, q_s and q_p are the dimensional surface charge densities of the solute and pore wall, respectively, R is the gas constant, ε_0 is the permittivity of free space, and ε_r is the dielectric constant of the solution. Expressions for the coefficients in equation (S1) are listed in Table S1.

Using the Helmholtz-Smoluchowski equation, q_p can be determined from the measured zeta potential on the membrane surface, ζ_p[1]:

$$q_p = 4\,CFk_D \sinh\left(\frac{F\zeta_p}{2RT}\right) \quad \text{(S4)}$$

The surface charge density on the spherical solute with radius r_s can be determined from the solute zeta potential ζ_s [2]:

$$q_s = \varepsilon_0\varepsilon_r \frac{\zeta_s}{r_s}\,(1 + k_D r_s) \quad \text{(S5)}$$

Table S1. Coefficients in the solution of the linearized Poisson Boltzmann equation (equations 6 and S1) where $\lambda = r_s / r_p$ and $\tau = r_p k_D^{-1}$ where r_s and r_p are the solute radius and the pore radius, and k_D is the Debye length. [3,4]

$\boldsymbol{A_s}$	$\boldsymbol{\frac{4\pi\tau\lambda^4 e^{\tau\lambda} S_0}{1+\tau\lambda}}$
$\boldsymbol{A_{sp}}$	$\frac{4\pi^2\lambda^2}{I_1(\tau)}$
$\boldsymbol{A_p}$	$\frac{\pi h(\tau\lambda)}{\tau^2 I_1^2(\tau)}$
$\boldsymbol{A_{den}}$	$\pi\tau(1+\tau\lambda)e^{-\tau\lambda} - S_0 h(\tau\lambda)$
	$h(\tau\lambda) = (1+\tau\lambda)e^{-\tau\lambda} - (1-\tau\lambda)e^{\tau\lambda}$
#	$S_0 = \int_0^{\infty} \frac{K_1[(\tau^2+\theta^2)^{1/2}]}{I_1[(\tau^2+\theta^2)^{1/2}]} d\theta$

Scanning Electron Microscopy Image of the Membrane Surface

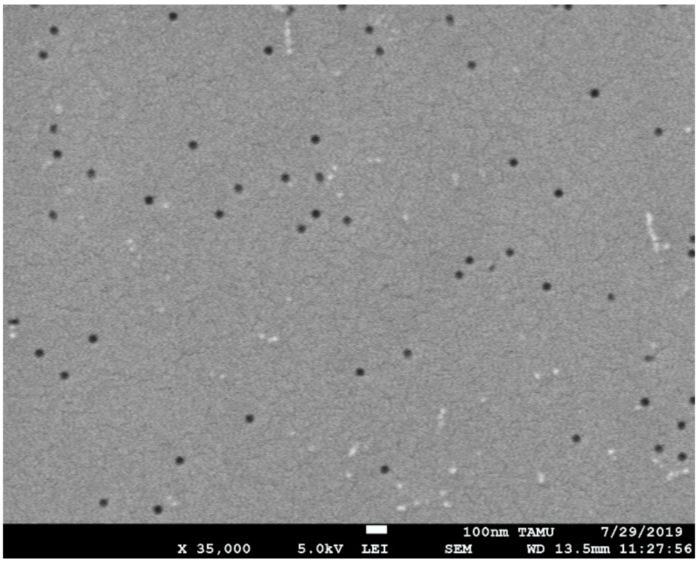

Figure S1 SEM image of membrane surface for 30 nm pore size membrane. Imaging was performed by the Chellam group at Texas A&M University

Table S2 Results from Filtration Experiments used to develop equation (8). The uncertainty values are standard deviations from permeate samples collected over time. Empirical model rejections are values calculated using equation (8).

Run	Pore size (nm)	TMP (psi)	NaCl Concentration (mM)	Rejection (%) (Experimental)	Rejection (%) (Empirical model)
1	30	10	0	97.1±1.7	85.8
2	30	20	7.5	50.9 ± 3.6	57.2
3	15	20	68.5	57.4 ± 4.1	56.0
4	15	30	56.5	58.5 ± 1.9	55.5
5	30	30	0	49.7 ± 7.9	49.6
6	10	30	100	79.1 ± 2.5	81.8
7	15	10	97.5	58.8 ± 2.0	60.6
8	30	20	60	22.8 ± 4.3	21.7
9	10	30	0	99.5 ± 0.4	100.0
10	15	20	68.5	61.6 ± 3.0	56.0
11	30	30	100	10.3 ± 2.6	7.7
12	15	30	56.5	58.4 ± 3.3	55.5
13	15	20	5	96.6 ± 1.4	90.1
14	10	20	36.5	85.6 ± 1.6	79.4
15	15	10	0	98.0 ± 1.4	99.2
16	10	10	31.5	75.4 ± 0.6	79.3
17	10	20	100	75.1 ± 1.9	79.4
18	30	20	60	13.4 ± 3.9	21.7

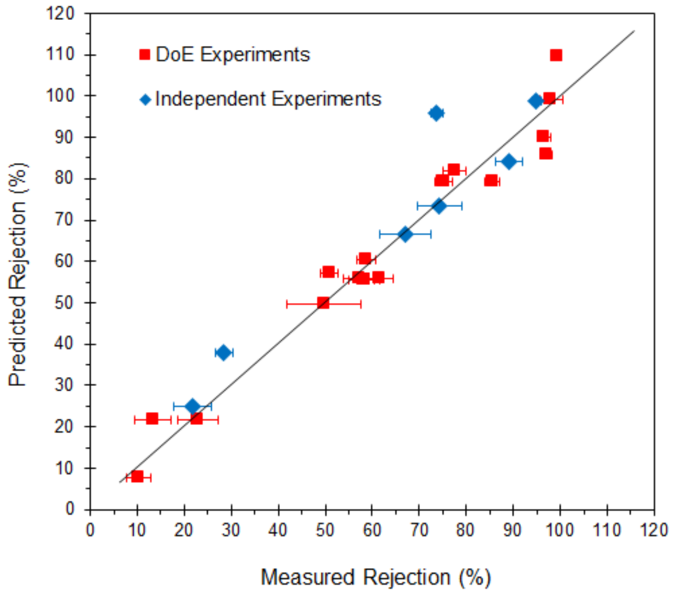

Figure S2 Parity plot of predicted vs. measured rejections. Predicted values are from equation (8). Data points include measured values from the eighteen experiments used to develop equation (8) (Table S2) as well as values from seven experiments that were not used in the model development. Error bars are the standard deviation from permeate samples collected over time for a single filtration experiment.

Hydrodynamic Radius as a function of NaCl concentration

Figure S3. shows the hydrodynamic radius of poly (dT_{60}) as a function NaCl concentration. A power law expression was fitted with the experimental data to estimate the size of DNA at various salt concentrations.

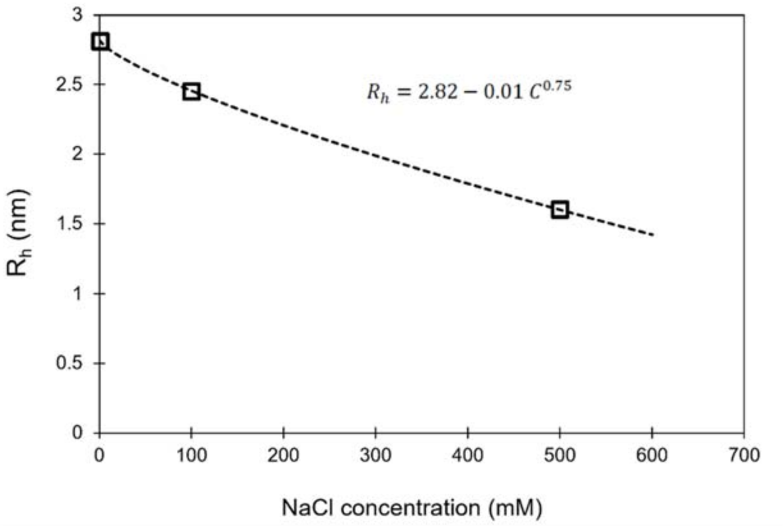

Figure S3. Hydrodynamic radius of poly (dT_{60}) ssDNA at different NaCl concentrations from the measured diffusion coefficients. The power law expression used to fit the experimental data is shown as a dashed line.

References

1. Rohani, M.M.; Zydney, A.L. Effect of Surface Charge Distribution on Protein Transport through Semipermeable Ultrafiltration Membranes. *Journal of Membrane Science* **2009**, *337*, 324–331, doi:10.1016/j.memsci.2009.04.007.

2. Mehta, A.; Zydney, A.L. Effect of Membrane Charge on Flow and Protein Transport during Ultrafiltration. *Biotechnology Progress* **2006**, *22*, 484–492, doi:10.1021/bp050324x.

3. Smith, F.G.; Deen, W.M. Electrostatic Double-Layer Interactions for Spherical Colloids in Cylindrical Pores. *Journal of Colloid and Interface Science* **1980**, *78*, 444–465, doi:10.1016/0021-9797(80)90584-6.

4. Burns, D.B.; Zydney, A.L. Contributions to Electrostatic Interactions on Protein Transport in Membrane Systems. *AIChE Journal* **2001**, *47*, 1101–1114, doi:https://doi.org/10.1002/aic.690470517.
